# Genome-Wide Analysis of the *FAR1*/*FHY3* (*FRS*) Gene Family and Expression Responses of *PbFRS* Genes to PEG-Induced Osmotic Stress, Light, and Shade in *Phoebe bournei*

**DOI:** 10.3390/ijms27115004

**Published:** 2026-06-01

**Authors:** Yizhuo Feng, Ronglin Liu, Ruobing Ying, Zekai Ding, Hengfeng Guan, Xinghao Tang, Kehui Zheng, Zhenzhen Zhang, Shijiang Cao

**Affiliations:** 1 College of Forestry, Fujian Agriculture and Forestry University, Fuzhou 350002, China; 12404028023@fafu.edu.cn (Y.F.); 52404022026@fafu.edu.cn (R.L.); 3236805016@stu.fafu.edu.cn (R.Y.); 3245320157@stu.fafu.edu.cn (Z.D.); hengfengguan777@gmail.com (H.G.); 2220428002@fafu.edu.cn (X.T.); 2 Fujian Academy of Forestry Sciences, Fuzhou 350012, China; 3 College of Computer and Information Sciences, Fujian Agriculture and Forestry University, Fuzhou 350002, China; zhkehui@fafu.edu.cn; 4 Fujian Provincial Key Laboratory of Haixia Applied Plant Systems Biology, Synthetic Biology Center, Haixia Institute of Science and Technology, Fujian Agriculture and Forestry University, Fuzhou 350002, China; zhenzhen-zhang@fafu.edu.cn

**Keywords:** *Phoebe bournei*, *FAR1*/*FHY3* (*FRS*) gene family, genome-wide analysis, PEG-induced osmotic stress, transcription factors, gene expression

## Abstract

Water availability and light conditions are among the most important environmental factors affecting tree growth and development. The *FAR1*/*FHY3* (*FRS*) gene family consists of transposase-derived transcription factors that are widely involved in light signaling and responses to environmental stresses. Although *FRS* genes have been characterized in several plant species, a comprehensive analysis in *P. bournei* is still lacking. In this study, we performed the first comprehensive genome-wide analysis of the *FRS* gene family in *P. bournei*, including physicochemical characterization, chromosomal localization, phylogenetic analysis, gene structure and conserved motif analysis, protein structure prediction, promoter cis-element analysis, organ/tissue expression profiling, and RT-qPCR analysis under PEG-induced osmotic stress, full-light, and shade treatments. A total of 21 *PbFRS* genes were identified and found to be unevenly distributed across 11 chromosomes. Phylogenetic analysis, together with *Arabidopsis thaliana* and *Zea mays* FRS proteins, clustered the family members into five clades, including one *P. bournei*-specific clade, suggesting lineage-specific expansion and possible functional diversification. Structural analyses revealed both conserved and divergent features among PbFRS members. Promoter analysis identified diverse cis-acting elements related to light, temperature, hormones, and stress responses, suggesting that *PbFRS* genes may have diverse regulatory potentials in response to environmental signals. Organ/tissue expression profiling further revealed clear differences in expression patterns among family members. In addition, RT-qPCR analysis showed that several genes, including *PbFRS9*, *PbFRS10*, *PbFRS12*, *PbFRS13*, *PbFRS16*, and *PbFRS18*, exhibited transcriptional responses to PEG-induced osmotic stress, full-light, and shade treatments. These results indicate that these genes may serve as candidates for future functional studies, although their direct roles in stress tolerance require further validation. Overall, these results provide the first systematic overview of the *PbFRS* gene family and identify transcriptionally responsive candidate genes for future functional studies in *P. bournei*.

## 1. Introduction

Plants are constantly exposed to changing environmental conditions throughout their life cycle. Given that they are sessile organisms, they cannot escape unfavorable environments and must rely on complex regulatory systems to perceive external signals and adjust their growth and development accordingly instead. Among multiple environmental conditions, water and light are two of the most important factors that determined plant survival, productivity, and geographical distribution. It is widely believed that water deficit could limit a series of normal growth behaviors of plants, such as cell expansion, photosynthesis, nutrient transport, and biomass accumulation. At the same time, unsuitable light conditions, including both excessive light and prolonged shading, can deform plant architecture, carbon assimilation, and stress adaptation. Therefore, elucidating the molecular basis of plant responses to water-deficit conditions and light-related signals is essential for understanding how transcriptional regulatory networks coordinate plant growth, development, and environmental responses [1,2,3].

Transcription factors (TFs) are central regulators of plant stress responses, and they can modulate the expression of downstream genes by recognizing specific cis-acting elements in promoter regions to help plants adapt to different environments. As the key nodes of regulatory networks, transcription factors integrate developmental cues with environmental signals and thereby coordinate physiological and morphological adaptation [1,4]. Among many transcription factor families identified in plants, the FAR-RED IMPAIRED RESPONSE 1/FAR-RED ELONGATED HYPOCOTYL 3 (*FAR1/FHY3*) family, also known as the FAR1-RELATED SEQUENCE (FRS) family, is particularly distinctive. Members of this family are believed to originate from Mutator-like element (MULE) transposases that were evolutionarily domesticated and subsequently acquired transcriptional regulatory functions [5,6,7]. In *A. thaliana* (L.), FRS proteins generally contain a conserved N-terminal DNA-binding domain, a central transposase-related region, and a C-terminal SWIM zinc-finger domain, which together contribute to DNA recognition, protein interaction, and transcriptional regulation [5,7]. Up to now, the typical structure of this significant family has been explored in model plants. Evolutionary analyses suggest that the FRS family underwent substantial diversification during plant evolution, implying that this family may have contributed to the establishment and expansion of regulatory networks in land plants [8].

As for functions, the FRS family is primarily recognized for its core role in light signaling. In *Arabidopsis*, FHY3 and FAR1 directly regulate the expression of FHY1 and FHL, thereby facilitating phytochrome A nuclear signaling and mediating far-red light responses [5,6]. Moreover, the functions of this family are not restricted to photomorphogenesis [9]. Increasing evidence has shown that FRS proteins are involved in a broad range of developmental and physiological processes, including circadian regulation, branching, leaf senescence, and the coordination of growth and defense [7,10,11,12,13]. For example, FHY3 and FAR1 have been shown to participate in leaf senescence regulation and shade-associated developmental responses, further highlighting the multifunctional nature of this family [12,13]. These findings indicate that FRS proteins act as important integrators of environmental and endogenous signals, rather than functioning solely as light-response regulators.

Beyond canonical light-signaling pathways, increasing evidence suggests that FRS members are associated with plant responses to abiotic environmental cues. In maize, several *ZmFRS* genes show differential expression under drought-related conditions, suggesting that they may be associated with transcriptional responses to water-deficit stress [14]. In Arabidopsis, FHY3 and FAR1 were proven that positively regulate ABA signaling and contribute to drought tolerance, in part by directly activating ABI5 expression [15]. In addition to drought, members of this family are involved in light-related stress and shade responses as well. In *Brassica napus* L., *FRS* genes exhibit differential expression under shading and low-temperature conditions [16]. In tomato, SlFHY3 has been shown to enhance cold tolerance under low red:far-red light conditions by cooperating with SlHY5, further supporting a role of FHY3/FAR1-related factors in environmental response regulation [17]. In *Arabidopsis*, FHY3 participates in shade avoidance signaling under low red:far-red light conditions, and together with FAR1 helps coordinate growth and defense responses under shade [13]. Collectively, current studies suggest that *FRS* family genes are involved in diverse light-related and abiotic stress responses across plant species. However, detailed functional evidence remains concentrated in *Arabidopsis* and a limited number of crops, whereas knowledge regarding *FRS* genes in woody plants, especially concerning their roles in long-term environmental adaptation, are still relatively scarce [18,19,20].

*Phoebe bournei* (Hemsl.) is an endemic and economically important timber tree species in China, and is a representative member of the Lauraceae. Owing to its desirable wood properties, including fine texture, fragrance, and decay resistance, it has long been valued for construction, high-quality furniture, and decorative wood products [21,22]. However, natural populations of *P. bournei* have declined sharply because of long-term exploitation and habitat disturbance [23]. Investigating the cause, drought and light limitation are the major environmental constraints affecting the growth, survival, and regeneration of *P. bournei* seedlings, thereby negatively impacting the plantation establishment and afforestation success [24,25,26,27]. With the availability of the *P. bournei* genome, genome-wide studies of environmentally responsive transcription factor families in this species have become possible, providing useful resources for understanding stress-related regulatory networks in woody plants [21,28]. Furthermore, several transcription factor families, including WRKY, GATA, and bZIP, have been identified and systematically analyzed in *P. bournei*, providing a foundation for understanding its stress-responsive regulatory networks [29,30,31]. However, the *FRS* family has not yet been systematically characterized in this species. In this study, we performed a genome-wide identification and analysis of the *FRS* gene family in *P. bournei*, including physicochemical characterization, chromosomal localization, phylogenetic analysis, conserved motif and gene-structure analysis, protein structure prediction, promoter cis-element analysis, organ/tissue expression profiling, and RT-qPCR analysis under PEG-induced osmotic stress, full-light, and shade treatments. Our findings provide the first comprehensive overview of the PbFRS family and identify transcriptionally responsive candidate genes for future functional studies on growth regulation and environmental responses in *P. bournei*.

## 2. Results

### 2.1. Identification and Physicochemical Analysis of PbFRS Gene Members

A total of 21 *PbFRS* genes were identified across the *P. bournei* genome through an integrated approach encompassing HMM searches and BLAST-v2.14.0 alignments. Then, using ProtParam, we gain further insights into the physicochemical properties of the FRS proteins (Table 1). Results showed that the 21 PbFRS proteins exhibit variation in the amino acid count, with length ranging from 78 to 825 residues, and the molecular weight of the proteins varied between 8.91 kDa (PbFRS5) and 93.02 kDa (PbFRS3). The isoelectric points spanned a range from 4.89 to 10.19, indicating that there were 6 acidic proteins (PI < 6.5), 3 neutral proteins (6.5 < PI < 7.5), and 12 basic proteins (PI > 7.5). Subcellular localization prediction suggested that 10 proteins were likely localized in the chloroplast and 9 in the nucleus, whereas the remaining proteins were predicted to be distributed in other organelles such as the Golgi apparatus and mitochondria, implying that functional differentiation may exist among the members. Furthermore, instability coefficient analysis unveiled that 6 proteins were classified as stable, whereas the remaining 15 proteins exhibited instability. Most PbFRS proteins were predicted to be unstable, whereas their isoelectric points indicated that the family contains both acidic and basic members, with basic proteins being more abundant.

### 2.2. Chromosome Localization Analysis of the PbFRS Gene Family

**Figure 1:**
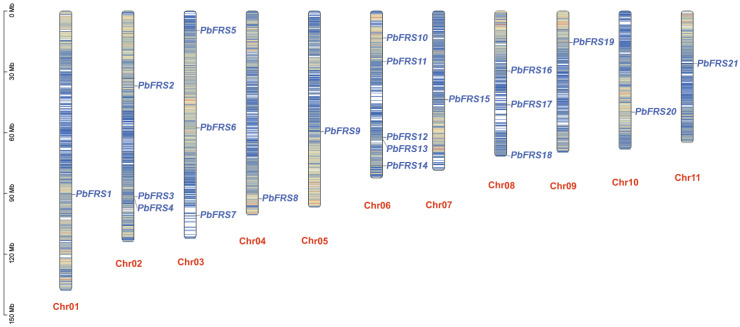
Chromosomal localization of the 21 *PbFRS* genes on 11 chromosomes of *P. bournei*; no *PbFRS* member was identified on chromosome 12. Chromosome size is presented in Mb, and the color gradient from blue to red indicates increasing gene density.

To determine the precise chromosomal localization of the *FRS* genes, we constructed a visualization of their distribution using MapChart-v6.6.1 software (Figure 1). The analysis results showed that the 21 *PbFRS* genes were unevenly distributed on 11 of the 12 chromosomes in *P. bournei*, with no PbFRS member detected on chromosome 12. Notably, chromosomes 6 exhibited the highest gene density, with a total of five genes, followed by chromosomes 2, 3, and 8, each containing three *PbFRS*s. Meanwhile, chromosomes 1, 4, 5, 7, 9, 10, and 11 each harbored only one *PbFRS* member. The chromosomal location of *PbFRS* genes suggests that this family may have undergone differential retention or expansion during genome evolution.

### 2.3. Display of the Motif and Gene Structure of PbFRS Gene Family

Members within the same subgroup usually shared similar motif types and motif orders, whereas the differences between subgroups were more obvious. This pattern suggests that the proteins may be relatively conserved within each subgroup, but more divergent among different subgroups.

**Figure 2:**
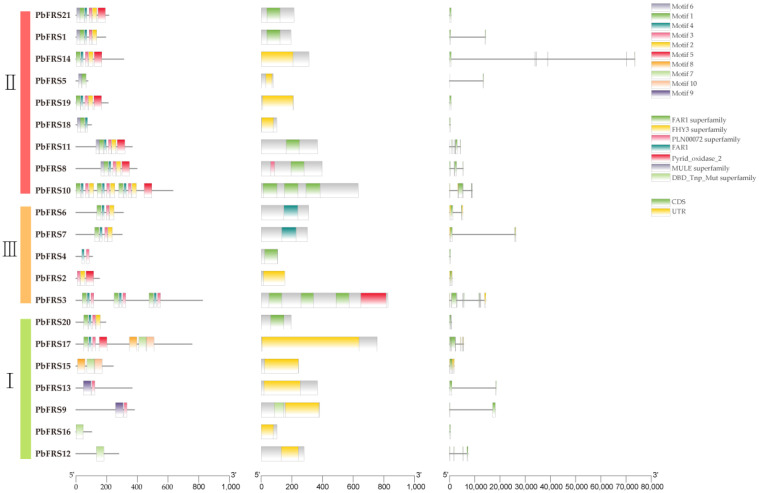
From left to right are the phylogenetic classification of *PbFRS* genes into three PbFRS-containing clades, conserved motif distribution, gene structure (CDS/UTR/introns), and domain distribution. All panels are aligned by gene order, with the horizontal axes representing sequence length and genomic coordinates, respectively.

All PbFRS proteins contained FAR1/FHY3-related domains, which supports their classification into this family (Figure 2). The domain organization also differed among clades. PbFRS10 in Clade II contained multiple tandem FAR1 domains. PbFRS3 in Clade III showed a rearranged domain pattern and also contained a pyridoxine oxidase domain. Several members in Clade I carried transposon-related domains such as MULE. This pattern may reflect the contribution of transposon-related events to family expansion.

Gene structure also varied among PbFRS members. Seven genes contained only CDS regions and lacked annotated UTRs, whereas the other members contained both CDS and UTR regions. The exon number differed among genes, but genes within the same subgroup were relatively similar. Taken together, these results show that the PbFRS family contains both conserved and variable structural features. The shared motifs and similar exon–intron patterns within subgroups suggest a certain degree of conservation, whereas the differences in motif composition, domain organization, and gene structure point to divergence among family members.

### 2.4. Phylogenetic Analysis of the PbFRS Gene Family

**Figure 3:**
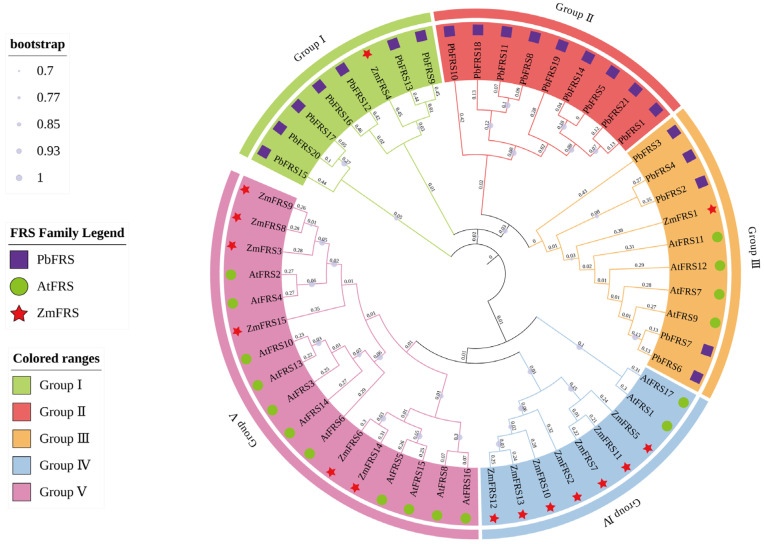
Phylogenetic tree of the *FRS* gene family in *P. bournei*, *A. thaliana* and *Z. mays*. Note: The phylogenetic tree was constructed based on the full-length FRS protein sequences of *P. bournei* (*PbFRS*, purple squares), *A. thaliana* (*AtFRS*, green circles) and *Z. mays* (*ZmFRS*, red stars), with bootstrap values to test the reliability of branches. All *FRS* genes were divided into five evolutionary groups (Group I–V), marked with different colored circular backgrounds. The numbers on the branches represent bootstrap support values, and the closer the value is to 1, the higher the reliability of the branch.

Phylogenetic analysis of FRS proteins from *P. bournei*, *A. thaliana*, and *Z. mays* clustered the sequences into five groups (Groups I–V) (Figure 3). The five groups differed in their species composition and degree of conservation.

Group I contained only one gene from *Z. mays*, whereas the other members were from *P. bournei*. Group II included only *PbFRS* genes and therefore represented a *P. bournei*-specific clade. This result suggests that some FRS members may have expanded specifically in this species. Group III contained members from all three species, which indicates that this group is relatively conserved. These genes may represent an older subset of the *FRS* family that formed before the divergence of monocots and dicots. Group IV was composed mainly of *Z. mays* genes together with a small number of *Arabidopsis* genes, but no *P. bournei* homologs were found in this group. This distribution suggests that this clade was retained or expanded differently in different lineages. Group V was shared by *A. thaliana* and *Z. mays* but did not contain any *P. bournei* members. This pattern may reflect gene loss or divergence in *P. bournei* during evolution.

The phylogenetic analysis suggests that the *FRS* gene family contains both conserved and lineage-specific members, in agreement with the patterns observed above. Conserved clades may preserve ancestral functions, whereas species-specific clades may represent diversification of the FRS family during the evolution of *P. bournei*.

### 2.5. Secondary and Tertiary Structure Prediction

Secondary-structure prediction revealed that PbFRS proteins were composed of four main elements, namely α-helices, extended strands, β-turns, and random coils, but their proportions varied considerably among family members (Table 2). Across the 21 PbFRS proteins, α-helices ranged from 14.81% to 70.87%, extended strands from 4.86% to 25.00%, β-turns from 1.65% to 10.19%, and random coils from 20.39% to 62.38%. On average, random coils accounted for the largest proportion (46.20%), followed by α-helices (34.99%), extended strands (14.22%), and β-turns (4.58%). PbFRS proteins contained both folded regions and flexible segments, but the proportions of these structural elements differed among family members. PbFRS5 (62.82%), PbFRS11 (60.76%), and PbFRS18 (62.38%) had relatively high random-coil contents, which may reflect greater structural flexibility. By contrast, PbFRS16 (70.87%), PbFRS15 (62.55%), PbFRS12 (55.56%), and PbFRS13 (50.82%) contained higher proportions of α-helices, which may be associated with more compact or ordered regions. PbFRS4 differed from most other members in that it showed the highest proportions of both extended strands (25.00%) and β-turns (10.19%). In general, proteins with similar secondary-structure compositions tended to show similar overall structural patterns, whereas differences in helix- and coil-rich regions were more obvious among some members.

**Figure 4:**
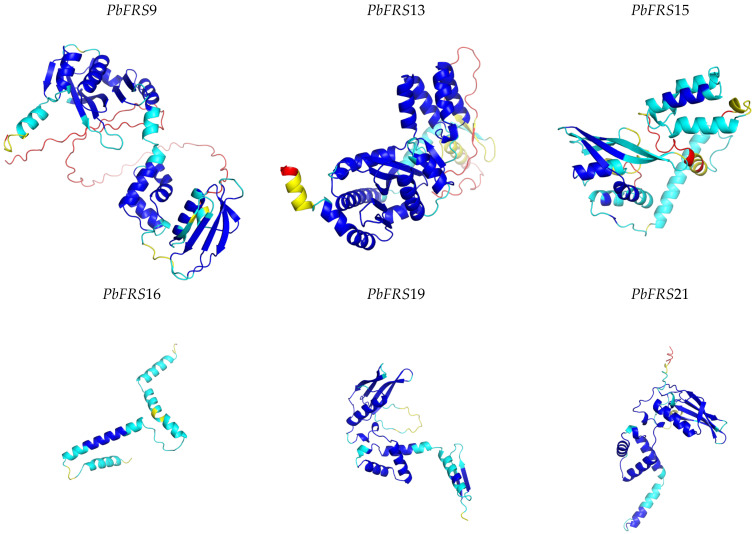
Tertiary structure models of six representative PbFRS proteins (PbFRS9, PbFRS13, PbFRS15, PbFRS16, PbFRS19, and PbFRS21). Colors represent pLDDT confidence scores: blue, very high; cyan, confident; yellow, low; red, very low.

We further predicted the tertiary structures of six representative proteins, namely PbFRS9, PbFRS13, PbFRS15, PbFRS16, PbFRS19, and PbFRS21, and visualized them in Figure 4. PbFRS9, PbFRS13, PbFRS19, and PbFRS21 showed relatively complete folded structures with high-confidence core regions. PbFRS15 and PbFRS16 also formed clear folded conformations, although some local regions had lower confidence. These models show both shared and variable features within the PbFRS family. Most representative proteins contained compact folded cores, while some also had flexible terminal or linker regions. Although only six proteins are shown in the main text, the predictions for other PbFRS members also supported the presence of conserved core regions together with variable flexible regions across the family.

### 2.6. Expression Profile Analysis of PbFRS Genes Across Different Organs/Tissues

**Figure 5:**
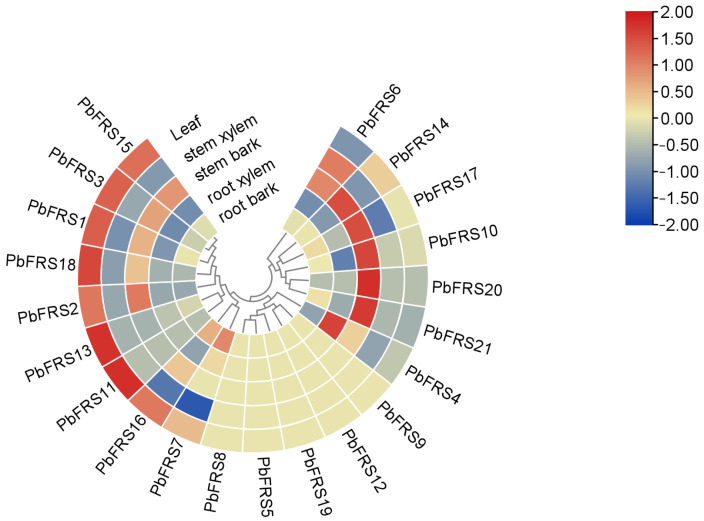
Heatmap showing the expression profiles of 21 *PbFRS* genes across five organs/tissues of *P. bournei*, including leaf, stem xylem, stem bark, root xylem, and root bark.

*PbFRS* gene expression was examined in five organs/tissues of *P. bournei*, including leaf, stem xylem, stem bark, root xylem, and root bark (Figure 5). The 21 genes showed clear differences in expression among these organs/tissues.

*PbFRS6*, *PbFRS10*, *PbFRS20*, and *PbFRS21* were expressed at relatively high levels in stem xylem. Some of these genes also showed comparatively high expression in stem bark. This pattern suggests that they may be related to stem development or vascular tissue formation in *P. bournei*. By contrast, *PbFRS4* and *PbFRS9* showed moderate expression in several tissues, which may indicate broader roles in growth and development. *PbFRS11*, *PbFRS13*, and *PbFRS15* were more highly expressed in leaves, suggesting that they may be associated with leaf-related processes, including light response.

These organ/tissue-specific expression patterns show clear variation among *PbFRS* members and provide useful information for future functional studies in *P. bournei*.

### 2.7. Synteny Analysis of FRS Genes in P. bournei, A. thaliana and O. sativa

**Figure 6:**
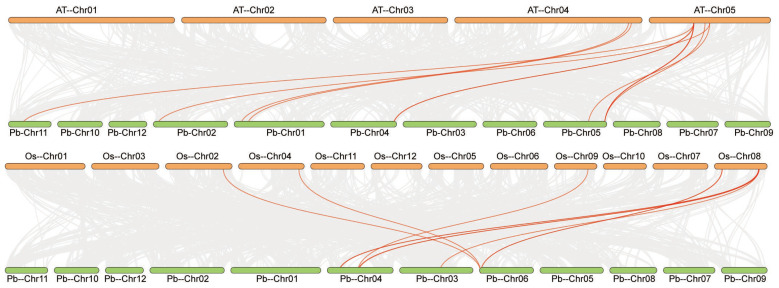
Syntenic relationships of *FRS* genes between *P. bournei* and the reference species *A. thaliana* and *O. sativa*. Gray lines indicate syntenic blocks between genomes, whereas red lines highlight collinear gene pairs involving *PbFRS* genes.

We performed genome-wide synteny analysis between *P. bournei* and two representative plant species, *A. thaliana* and *O. sativa*, to examine the conservation and homologous relationships of *FRS* genes across species (Figure 6). In the comparison with *A. thaliana*, eight *PbFRS* genes showed syntenic relationships with homologous genes in this species. These genes were mainly located on chromosomes 1, 4, and 5 of *P. bournei*, and chromosome 5 contained the largest number of them. In addition, chromosome 5 of *A. thaliana* showed syntenic links with multiple *P. bournei* chromosomes, which may indicate that this region retained more FRS homologs.

In the comparison with *O. sativa*, seven *PbFRS* genes showed syntenic relationships with homologous genes in rice. These genes were mainly distributed on chromosomes 4 and 6 of *P. bournei*. Chromosome 8 of *O. sativa* also showed syntenic links with multiple *P. bournei* chromosomes, which may reflect the retention of more FRS homologs in this region. Overall, the two comparisons showed similar numbers of syntenic gene pairs, which suggests that part of the FRS family has remained conserved during angiosperm evolution.

### 2.8. Cis-Acting Elements Analysis of PbFRS Genes

**Figure 7:**
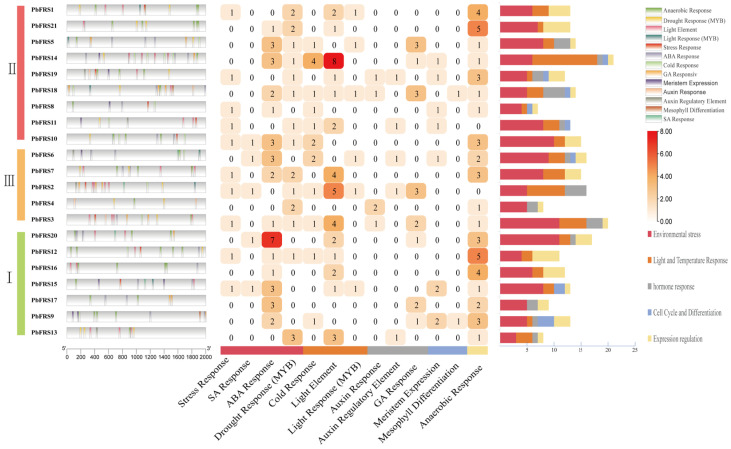
Analysis of cis-acting elements in promoters of *PbFRS* genes in *Phoebe bournei*. From left to right: distribution, quantity heatmap and functional classification of cis-acting elements. Elements were classified into five categories: environmental stress response, light and temperature response, hormone response, cell cycle and differentiation, and expression regulation.

To elucidate the regulatory characteristics of *PbFRS* genes, cis-acting elements in the 2000 bp promoter regions of the 21 *PbFRS* genes were identified and classified (Figure 7). The cis-elements found in this gene family could be grouped into five categories: environmental stress response, light and temperature response, hormone response, cell cycle and differentiation, and expression regulation. Among them, environmental stress response and light–temperature response elements were relatively abundant, which is consistent with the reported roles of *FAR1*/*FHY3* family members in light signaling and stress responses.

Because PbFRS members were distributed only in Groups I–III of the combined phylogenetic tree, cis-element distribution was further compared among these three PbFRS-containing clades. Genes in Clade II were relatively enriched in light–temperature response elements and environmental stress response elements. For example, *PbFRS14* contained eight light-responsive elements. This result suggests that genes in this clade may be closely related to environmental signal responses. Genes in Clade III were enriched in both light–temperature response elements and hormone response elements. *PbFRS2*, as a representative member of this clade, contained five light-responsive elements and three hormone-responsive elements. This pattern indicates that members of Clade III may respond to both light-related and hormone-related signals. In Clade I, environmental stress response elements were also common. In addition, some genes, such as *PbFRS13*, were enriched in cell differentiation-related elements, whereas *PbFRS20* contained seven salicylic acid-responsive elements. These differences suggest that members of Clade I may be involved in multiple regulatory pathways.

The promoter analysis indicates that *PbFRS* genes may possess diverse regulatory potentials and that different clades may have undergone regulatory divergence during evolution. These results provide useful clues for selecting candidate *PbFRS* genes for future studies on light-related signaling, environmental responses, and plant growth and development.

### 2.9. Expression Analysis of Candidate PbFRS Genes Under PEG-Induced Osmotic Stress, Full-Light, and Shade Treatments

**Figure 8:**
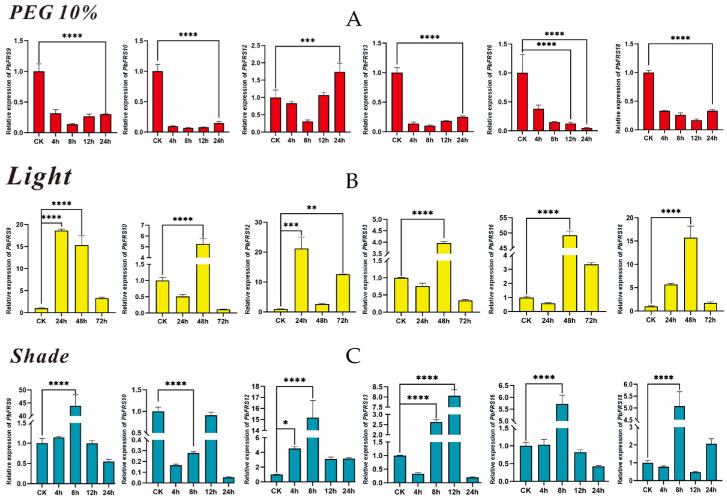
Expression profiles of *PbFRS* genes under PEG-induced osmotic stress, full-light, and shade treatments, as determined by RT-qPCR. (**A**) Relative expression levels of *PbFRS* genes under 10% PEG6000-induced osmotic stress at 4, 8, 12, and 24 h. (**B**) Relative expression levels of *PbFRS* genes under full-light treatment at 24, 48, and 72 h. (**C**) Relative expression levels of *PbFRS* genes under shade treatment (30% of full light) at 4, 8, 12, and 24 h. Samples collected at 0 h under normal growth conditions before stress treatment were used as controls. Different time points within each treatment were analyzed by one-way ANOVA followed by Dunnett’s multiple comparisons test, using the 0 h sample as the control. Asterisks indicate significant differences compared with the 0 h control (* *p* < 0.05, ** *p* < 0.01, *** *p* < 0.001, **** *p* < 0.0001).

Based on promoter cis-element analysis and organ/tissue expression profiling, six *PbFRS* genes (*PbFRS9*, *PbFRS10*, *PbFRS12*, *PbFRS13*, *PbFRS16*, and *PbFRS18*) were selected as representative candidates for RT-qPCR analysis. The promoter regions of these genes were relatively enriched in cis-elements associated with environmental responses and light-related signals, and some of them also showed detectable transcript abundance in leaves, supporting their use in subsequent leaf-based expression analysis. Subsequently, their transcript levels under PEG-induced osmotic stress, full-light, and shade treatments were analyzed using RT-qPCR. Under 10% PEG6000-induced osmotic stress, all six candidate genes exhibited transcriptional responses to the treatment (Figure 8A). Overall, their expression levels showed a dynamic trend of initially decreasing and then increasing over time: *PbFRS9*, *PbFRS10*, *PbFRS12*, and *PbFRS13* reached their lowest expression levels at 8 h, followed by a slight upward trend, whereas the expression level of *PbFRS16* continuously decreased over time.

Under full-light treatment, *PbFRS9* and *PbFRS18* were upregulated at the early stage and then decreased at later time points (Figure 8B). By contrast, *PbFRS10*, *PbFRS13*, and *PbFRS16* showed an opposite pattern, with an initial decrease followed by an increase that peaked at 48 h and then declined. *PbFRS12* displayed a more fluctuating pattern, increasing at first, then decreasing, and rising again thereafter.

Under shade treatment conditions, the expression levels of *PbFRS9*, *PbFRS12*, *PbFRS16*, and *PbFRS18* generally showed a trend of first increasing and then decreasing, peaking at 8 h (Figure 8C). *PbFRS13* also showed a clear transcriptional response to shade treatment, although its temporal pattern differed slightly from those of the four genes above. In contrast, the expression level of *PbFRS10* exhibited a pattern of initially decreasing, then increasing, and finally declining. All six genes showed significant differences in expression under full-light, shade, and 10% PEG6000-induced osmotic stress treatments, with both upregulation and downregulation observed. These results indicate that several PbFRS members are transcriptionally responsive to PEG-induced osmotic stress and altered light conditions in *P. bournei*.

## 3. Discussion

In this study, 21 *PbFRS* genes were identified from the *P. bournei* genome. This number suggests that the *FRS* family has undergone a certain degree of expansion in this species, although the extent of expansion is not unusually large when compared with that reported in other plants. Similar genome-wide analyses in tea plant, rapeseed, and maize have also shown that the number and composition of *FAR1*/*FHY3* family members differ among species [14,16,19].In Arabidopsis, FHY3 and FAR1 were first identified as transcription factors derived from Mutator-like transposases, which provided key evidence for the evolutionary origin of this family [5]. Later studies further showed that *FHY3/FAR1* are involved not only in phyA-mediated light signaling, but also in development and abiotic stress responses [6,7,32]. In our study, the obvious differences in amino acid length, molecular weight, isoelectric point, instability index, and predicted subcellular localization among PbFRS proteins also point to considerable diversification within this family.

The phylogenetic and synteny results further showed that the *PbFRS* family contains both conserved members and species-specific components. The FRS proteins from *P. bournei*, *A. thaliana*, and *Z. mays* were divided into five clades, and Group II contained only *P. bournei* members. This result suggests that at least part of the FRS family may have expanded specifically in this woody species. By contrast, Group III included members from all three species, which implies that this subgroup is relatively conserved and may have originated before the divergence of monocots and dicots. Some other clades showed unequal representation, or even the absence, of *P. bournei* members, which may reflect gene gain, loss, or divergence during evolution. Similar subgroup differentiation has also been reported in tea plant, rapeseed, maize, and peanut [16,19,33,34]. The synteny analysis was consistent with the phylogenetic pattern. Eight *PbFRS* genes showed collinear relationships with homologs in *A. thaliana*, and seven showed syntenic relationships with homologs in *O. sativa*. These results suggest that part of the FRS family has been retained in conserved genomic regions across angiosperms. Members within the same *PbFRS* subgroup also tended to share similar motif composition and exon-intron organization. This consistency suggests that some structural and functional features may have been preserved during evolution [35]. In *P. bournei*, the presence of a species-specific clade is notable. As a long-lived woody tree, *P. bournei* is exposed to repeated changes in light conditions and seasonal water limitation. The expansion of some *PbFRS* members may have increased regulatory flexibility in this species, although direct functional evidence is still needed.

The structural analyses showed a similar pattern. Most PbFRS proteins were composed mainly of α-helices and random coils, whereas extended strands and β-turns accounted for smaller proportions. This pattern suggests that the family shares some common folding features, although clear differences were also present among members. The tertiary-structure prediction showed the same trend. Several representative proteins, especially PbFRS13, PbFRS19, and PbFRS21, contained relatively complete folded cores with high confidence, whereas some other members appeared to have more flexible terminal or linker regions. These differences may be related to functional divergence within the family, although the current structural predictions should be regarded as supportive rather than definitive evidence. Previous studies on Arabidopsis FHY3/FAR1 have shown that different conserved domains contribute differently to DNA binding and signaling functions [5,6,7]. Based on this, the coexistence of conserved domains and local structural variation in PbFRS proteins may be associated with both shared and distinct roles.

The promoter and expression analyses also point to differences among *PbFRS* genes. The promoters contained cis-elements related to light, temperature, hormone, and stress responses, which is broadly consistent with previous reports for *FRS* family members in other species [16,19,36]. The organ/tissue expression data showed additional variation. Several *PbFRS* genes were preferentially expressed in stem xylem or stem bark, whereas others were more highly expressed in leaves. This pattern suggests that different members may play different spatial roles during growth and development. Similar organ- or tissue-biased expression patterns have also been reported in other *FAR1/FHY3* family studies [37,38]. In our dataset, the promoter analysis, tissue-expression patterns, and RT-qPCR results were generally consistent with one another. Genes with more light-, temperature-, or stress-related cis-elements tended to show clearer transcriptional responses under PEG-induced osmotic stress, full-light, or shade treatments. These results do not prove gene function on their own, but they do suggest that at least some *PbFRS* members may be associated with transcriptional regulation under environmental treatments in *P. bournei*.

The RT-qPCR results further demonstrated that six candidate genes—*PbFRS9*, *PbFRS10*, *PbFRS12*, *PbFRS13*, *PbFRS16*, and *PbFRS18*—responded differentially to PEG-induced osmotic stress, full-light, and shade treatments. These divergent expression patterns indicate that *PbFRS* genes may be transcriptionally regulated by osmotic and light-related environmental cues. Similar expression-level responses of *FAR1*/*FHY3* family genes have been reported in multiple plant species. In maize, several *FAR1*/*FHY3* genes showed differential expression under drought-related treatments [14]. In rapeseed, several *BnFAR1*/*FHY3* genes responded to shading treatment, and their promoters contained cis-elements related to light and abiotic stress responses [16]. In tea plants, *CsFHY3*/*FAR1* genes were also found to respond to multiple abiotic conditions, further supporting the association of this family with environmental responses [19]. Moreover, previous mechanistic studies and reviews in Arabidopsis have shown that *FHY3*/*FAR1* can integrate light signaling with ABA-related pathways [6,32]. Therefore, the present RT-qPCR data suggest that selected *PbFRS* genes are transcriptionally responsive to PEG-induced osmotic stress and altered light conditions. However, these expression changes should not be interpreted as direct evidence that *PbFRS* genes confer stress tolerance.

Overall, this study provides the first systematic overview of the *FRS* family in *P. bournei*. The results show that the *PbFRS* family contains both conserved structural features and species-specific evolutionary characteristics. The combined evidence from phylogenetic analysis, motif and gene-structure analysis, structural prediction, promoter cis-element analysis, organ/tissue expression profiling, and RT-qPCR analysis suggests that some *PbFRS* genes are transcriptionally responsive to PEG-induced osmotic stress and altered light conditions. It should be noted that changes in transcript abundance alone cannot demonstrate that these genes directly confer stress tolerance. In addition, it should also be noted that the subcellular localization information in this study was obtained from bioinformatic prediction. Although these predictions provide useful preliminary information for understanding possible functional divergence among PbFRS proteins, they do not represent experimental localization evidence. Therefore, transient expression assays in tobacco epidermal cells or other experimental systems will be needed in future studies to confirm the precise subcellular localization of PbFRS proteins. Plant water status was not directly measured in the present study. Therefore, the PEG6000 treatment should be interpreted as PEG-induced osmotic stress rather than natural drought. Future studies should include measurements of relative water content, leaf water potential, osmotic adjustment, ROS accumulation, and photosynthetic performance to evaluate the possible relationship between *PbFRS* gene expression and water-deficit responses in *P. bournei*. Further genetic and molecular analyses, including loss- or gain-of-function studies, will also be needed to clarify the precise biological roles of *PbFRS* genes in *P. bournei*.

## 4. Materials and Methods

### 4.1. Identification of FAR1/FHY3 (FRS) Family Members in P. bournei and Physicochemical Characterization

The whole-genome sequence and annotation files of *P. bournei* were obtained from the China National GeneBank DataBase (CNGBdb) under accession number CNP0002030. To identify members of the *FAR1/FHY3 (FRS)* gene family in *P. bournei*, 17 Arabidopsis thaliana FAR1-family protein sequences were first downloaded from PlantTFDB (https://planttfdb.gao-lab.org/family.php?sp=Ath&fam=FAR1, accessed on 26 November 2025). Based on the genome annotation of *P. bournei*, coding sequences (CDSs) were extracted and translated into protein sequences using TBtools-II v2.388. These *A. thaliana* FAR1 protein sequences were then used as queries to perform a BLASTP search against the predicted *P. bournei* protein dataset, and the annotation information of candidate sequences was further checked using the NCBI database. Putative FRS family members were preliminarily screened based on sequence similarity and annotation results. To further confirm candidate genes, Hidden Markov Model (HMM) profiles of *FAR1/FHY3*-related conserved domains, including PF03101, PF04434, and PF10551, were downloaded from the Pfam database and used as queries to search all predicted protein sequences of *P. bournei* with HMMER v3.3.2, using an E-value threshold of 1 × 10^−5^ [39]. Candidate proteins were subsequently verified using the Conserved Domain Database (CDD) of NCBI and SMART [40,41]. Redundant sequences and proteins lacking characteristic FAR1/FHY3-related conserved domains were manually removed. Finally, 21 non-redundant *FAR1/FHY3* family members were identified in the *P. bournei* genome.

The annotated genome of *P. bournei* was used to determine the chromosomal coordinates of all identified *PbFRS* genes, and their distribution was plotted with MapChart v2.32. Gene names (*PbFRS1*-*PbFRS21*) were assigned on the basis of chromosomal location. Basic protein features, including sequence length, molecular weight, theoretical pI, instability index, aliphatic index, and GRAVY value, were estimated using ExPASy ProtParam [42], while the putative subcellular localization of PbFRS proteins was predicted using WoLF PSORT [43].

### 4.2. Conserved Motif, Domain, and Exon–Intron Structure Analysis of PbFRS Genes

Conserved motifs in the 21 PbFRS proteins were analyzed with MEME v5.5.9, using a motif width range of 6–50 amino acids [44]. Ten motifs were detected in total, and their distribution was visualized using TBtools v2.388 [45]. Conserved domains were further examined using CDD and SMART. All PbFRS proteins contained at least one typical FAR1/FHY3-related conserved domain, including the FAR1 DNA-binding domain (PF03101), the SWIM zinc-finger domain (PF04434), and the MULE transposase-like domain (PF10551). Exon–intron structures of the PbFRS genes were analyzed using GSDS2.0 [46]. Structural domains were also examined using the Batch CD-search tool of NCBI [47,48]. Phylogenetic relationships, conserved motif composition, domain organization, and exon-intron structures were integrated and visualized using the Gene Structure View function of TBtools.

### 4.3. Phylogenetic Analysis of PbFRS Proteins

The coding DNA sequences (CDS), protein sequences, and genomic sequences of *Z. mays FAR1*/*FHY3* family members were obtained from the Grassius database (https://grassius.org/species/Maize/ (accessed on 26 November 2025)). To clarify the evolutionary relationships of *FAR1*/*FHY3* family proteins from *P. bournei*, *A. thaliana*, and *Z. mays*, phylogenetic analysis was conducted using MEGA11 [49]. The phylogenetic tree was generated by the neighbor-joining (NJ) method with default settings and 1000 bootstrap replicates. Tree visualization and annotation were performed using iTOL (https://itol.embl.de/ (accessed on 26 November 2025)) [50]. Bootstrap values were used to assess branch reliability, and major clades were labeled according to the subgroup classification of the *PbFRS* family.

### 4.4. Analysis of Organ/Tissue Expression Profiles of PbFRS Genes

Organ/tissue expression patterns of *PbFRS* genes were examined using publicly available RNA-seq data from *P. bournei*. The dataset (accession no. PRJNA628065) was retrieved from the EMBL-EBI repository (https://www.ebi.ac.uk/ena/browser/view/PRJNA628065/ (accessed on 26 November 2025)). Transcript abundance of *PbFRS* genes was obtained from five organs/tissues and represented as FPKM (Fragments Per Kilobase Million) values. After log2 transformation, the data were visualized as a heatmap using TBtools v2.388 to compare expression differences among organs/tissues.

### 4.5. Synteny Analysis of PbFRS Genes

To examine the evolutionary conservation of *PbFRS* genes, comparative collinearity analysis was performed using *A. thaliana* and *O. sativa* as reference species. Genome sequence files for the two species were obtained from EnsemblPlants (https://plants.ensembl.org/index.html/ (accessed on 26 November 2025)) and Phytozome v13 (https://phytozome-next.jgi.doe.gov/ (accessed on 26 November 2025)), respectively. Syntenic relationships between *PbFRS* genes and their corresponding homologs were analyzed and visualized with the Synteny Visualization function in TBtools v2.388. The resulting collinear blocks were used to assess evolutionary conservation and possible functional divergence within the *FAR1*/*FHY3* gene family.

### 4.6. Secondary and Tertiary Structure Prediction of PbFRS Proteins

Secondary structures of PbFRS proteins were predicted using the SOPMA online server [51]. The proportions of α-helices, extended strands, β-turns, and random coils were calculated for each protein and compared among PbFRS family members.

To further characterize the three-dimensional structures of PbFRS proteins, representative proteins were submitted to the ColabFold notebook (v1.6.1) on the Google Colab platform. This platform uses AlphaFold2 for protein structure prediction (https://colab.research.google.com/github/sokrypton/ColabFold/blob/main/AlphaFold2.ipynb?pli=1#scrollTo=kOblAo-xetgx/ (accessed on 20 March 2026)) [52,53,54]. Protein sequences were submitted individually using the default monomer prediction mode, and multiple sequence alignments were generated using the MMseqs2-based pipeline. Model quality was evaluated using predicted local distance difference test (pLDDT) scores and predicted aligned error (PAE) values. Representative proteins with relatively reliable models were selected for structural visualization and comparative analysis. The predicted structures were displayed using the pLDDT color scheme, in which warmer colors indicate lower confidence and cooler colors indicate higher confidence.

### 4.7. Cis-Acting Element Analysis of PbFRS Gene Promoters

Promoter cis-acting elements of *PbFRS* genes were analyzed using the GTF/GFF3 Sequences Extract function in TBtools v2.388. A 2000 bp upstream region of each *PbFRS* gene was extracted from the *P. bournei* genome and used for promoter analysis. Cis-acting elements were predicted using PlantCARE [36]. The identified elements were classified into five categories: environmental stress response, light and temperature response, hormone response, cell cycle and differentiation, and expression regulation. The distribution and frequency of these cis-elements were organized and visualized using TBtools v2.388.

### 4.8. Plant Materials and Treatment Conditions

One-year-old *P. bournei* seedlings (germplasm number: Jian’ou No. 8) obtained from the Fujian Academy of Forestry were used for treatment experiments. Seedlings were grown in pots containing a mixture of peat soil, humus soil, sandy soil, and perlite (5:2:2:1, *v*/*v*/*v*/*v*) under controlled conditions in an artificial climate chamber with a 12 h light/12 h dark photoperiod at 25 °C. The normal growth condition was set at 60% of full light. For stress treatments, seedlings with uniform growth were divided into different treatment groups. PEG-induced osmotic stress was applied using nutrient solution supplemented with 10% PEG6000. This concentration was selected to impose a moderate short-term osmotic stress suitable for transcriptional response analysis while avoiding severe tissue damage during the sampling period. The osmotic potential of the 10% PEG6000 solution was estimated according to published empirical equations for PEG solutions. The full-light treatment was set at 100% chamber light intensity, corresponding to a photosynthetic photon flux density (PPFD) of 1200 μmol·m^−2^·s^−1^. The shade treatment was imposed at 30% of full light, corresponding to a PPFD of 360 μmol·m^−2^·s^−1^. Leaf samples were collected at 0, 4, 8, 12, and 24 h for PEG-induced osmotic stress and shade treatments, and at 0, 24, 48, and 72 h for full-light treatment. Three independent biological replicates were collected for each treatment and time point, and each biological replicate consisted of mixed leaves from two seedlings. Samples collected at 0 h, before stress treatment, under normal growth conditions were used as the controls for relative expression analysis.

### 4.9. Real-Time Quantitative Polymerase Chain Reaction (RT-qPCR) Analysis of PbFRS Genes

Total RNA was extracted from leaf samples using the HiPure Plant RNA Mini Kit (Magenbio, Guangzhou, China). First-strand cDNA was synthesized using the PrimeScript RT Reagent Kit (Perfect Real Time) (TaKaRa, Dalian, China). For RT-qPCR analysis, six representative *PbFRS* genes (*PbFRS9*, *PbFRS10*, *PbFRS12*, *PbFRS13*, *PbFRS16*, and *PbFRS18*) were selected based on promoter cis-element composition and organ/tissue expression information. Priority was given to genes whose promoter regions were enriched in cis-elements associated with environmental and light-related responses and that showed detectable expression in leaves. Primer specificity and the feasibility of leaf-based expression detection were also considered during the final selection. Gene-specific primers were designed in non-conserved regions using Primer 3.0 software, and the primer sequences are listed in Table 3.

Quantitative RT-PCR was performed in a total volume of 20 μL, including 1 μL cDNA template, 10 μL SYBR Premix Ex Taq II, 2 μL gene-specific primers, and 7 μL ddH2O. Cycling conditions consisted of an initial denaturation at 95 °C for 30 s, followed by 40 amplification cycles at 95 °C for 5 s and 60 °C for 30 s. PbEF1α (GenBank No. KX682032) was used as the reference gene, and relative expression levels were calculated with the 2^−ΔΔCt^ method [55,56]. Statistical analysis was performed using GraphPad Prism 10.1.2. For each treatment, expression differences among time points were assessed by one-way ANOVA with Dunnett’s multiple comparisons test, taking 0 h as the control. A value of *p* < 0.05 was regarded as statistically significant.

## 5. Conclusions

In this study, 21 *FAR1*/*FHY3* family genes were identified in the *P. bournei* genome, and their physicochemical properties, chromosomal distribution, phylogenetic relationships, conserved motifs, gene structures, secondary and tertiary structures, promoter cis-elements, and organ/tissue expression profiles were systematically analyzed. The results revealed both conserved and divergent characteristics within the *PbFRS* family. Promoter analysis showed that many *PbFRS* genes contain cis-elements related to light, hormone, temperature, and stress-related responses. RT-qPCR analysis further showed that six selected *PbFRS* genes exhibited transcriptional responses to PEG-induced osmotic stress, full-light, and shade treatments. These findings provide a useful genomic and expression-level resource for the *PbFRS* family and identify candidate genes for future functional studies. However, the present results do not establish a direct causal relationship between *PbFRS* gene expression and stress tolerance. Further physiological, genetic, and molecular analyses will be required to clarify the biological functions of *PbFRS* genes in *P. bournei*.

## Figures and Tables

**Table 1:** Physical and chemical properties of PbFRS family members.

Gene ID	Gene Name	Molecular Weight (kDa)	Number of Amino Acids	Theoretical PI	Instability Index	Aliphatic Index	GRAVY	Predicted Subcellular Localization
OF22842	PbFRS1	22.723	194	8.36	56.22	41.13	−1.081	Nucleus
OF18844	PbFRS2	17.62	153	9.51	37.23	82.09	−0.461	Chloroplast
OF23050	PbFRS3	93.02	825	6.68	35.73	69.68	−0.632	Nucleus
OF23061	PbFRS4	12.21	108	10.19	55.7	80.37	−0.364	Chloroplast
OF13205	PbFRS5	8.91	78	5.41	28.24	74.87	−0.453	Chloroplast
OF24153	PbFRS6	35.23	309	5.14	62.22	56.47	−1.138	Nucleus
OF14738	PbFRS7	34.37	301	5.47	60.49	70.63	−0.888	Chloroplast
OF01678	PbFRS8	45.12	398	8.87	38.41	64.37	−0.67	Nucleus
OF02678	PbFRS9	44.09	381	9.00	35.2	83.67	−0.318	Nucleus
OF18405	PbFRS10	72.96	632	9.41	54.14	57.67	−0.971	Nucleus
OF00915	PbFRS11	41.07	367	6.37	44	63.43	−0.618	Chloroplast
OF07214	PbFRS12	32.01	279	6.52	43.37	73.76	−0.413	Chloroplast
OF28777	PbFRS13	41.64	366	9.08	42.39	84.04	−0.324	Chloroplast
OF29536	PbFRS14	35.94	311	10.02	44.49	60.45	−0.858	Mitochondrion
OF15437	PbFRS15	28.68	243	5.43	37.62	78.15	−0.363	Nucleus
OF05523	PbFRS16	12.10	103	9.17	55.71	69.03	−0.417	Nucleus
OF10408	PbFRS17	87.76	756	7.09	53.6	71.03	−0.616	Chloroplast
OF14737	PbFRS18	11.75	101	9.48	43.81	60.69	−0.662	Chloroplast
OF02972	PbFRS19	24.22	210	8.91	46.72	80.19	−0.527	Nucleus
OF20764	PbFRS20	22.20	195	4.89	62.2	78.46	−0.684	Chloroplast
OF15101	PbFRS21	24.99	214	9.43	53.4	49.16	−1.057	Golgi apparatus

**Table 2:** Predicted secondary-structure composition of PbFRS proteins.

Protein	Proportions of Secondary-Structure Elements (%)				Schematic Representation of Predicted Secondary Structures
	α-Helix	Extended Strand	β-Turn	Random Coil	
PbFRS1	33.51	16.49	5.15	44.85	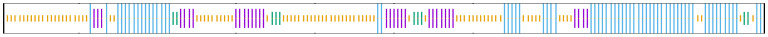
PbFRS2	34.64	17.65	7.19	40.52	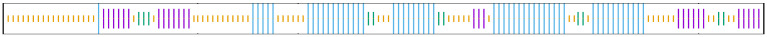
PbFRS3	33.33	15.27	2.79	48.61	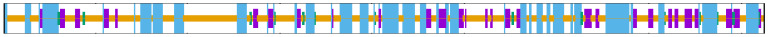
PbFRS4	14.81	25.00	10.19	50.00	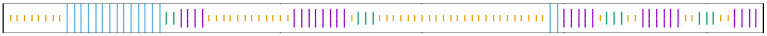
PbFRS5	19.23	15.38	2.57	62.82	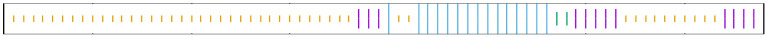
PbFRS6	30.10	9.39	3.88	56.63	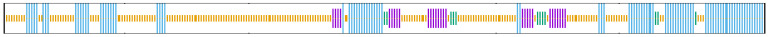
PbFRS7	29.90	10.63	2.33	57.14	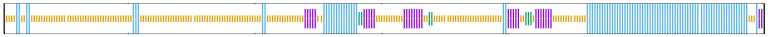
PbFRS8	29.15	7.54	3.76	59.55	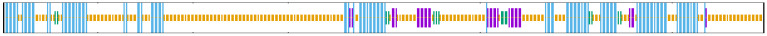
PbFRS9	32.28	17.59	3.94	46.19	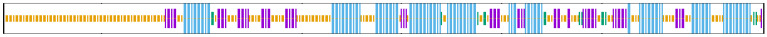
PbFRS10	34.81	16.93	4.91	43.35	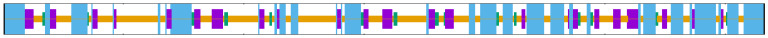
PbFRS11	22.89	12.26	4.09	60.76	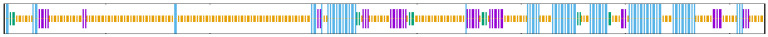
PbFRS12	55.56	10.39	3.23	30.82	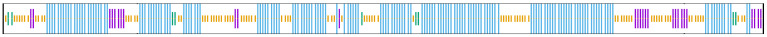
PbFRS13	50.82	15.30	3.83	30.05	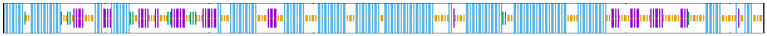
PbFRS14	33.12	19.94	6.43	40.51	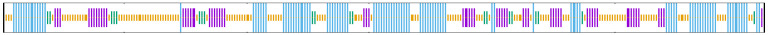
PbFRS15	62.55	7.82	1.65	27.98	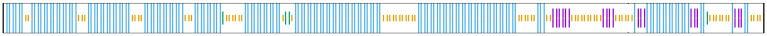
PbFRS16	70.87	4.86	3.88	20.39	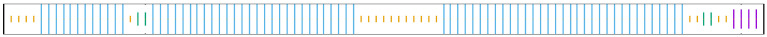
PbFRS17	41.93	9.39	2.65	46.03	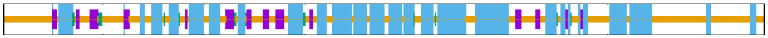
PbFRS18	15.84	17.82	3.96	62.38	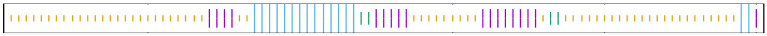
PbFRS19	34.29	18.57	7.14	40.00	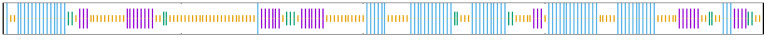
PbFRS20	22.05	15.90	6.15	55.90	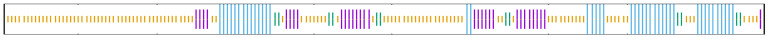
PbFRS21	33.18	14.49	6.54	45.79	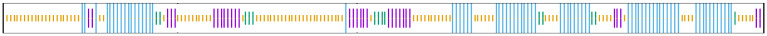

**Table 3:** Primer sequences of quantitative RT-qPCR.

Primer	Sequence (5′~3′)
PbFRS9 (OF02678-F)	GCGGTCGACGGATCACAT
PbFRS9 (OF02678-R)	CCACTCCCAGCTGTCACG
PbFRS10 (OF18405-F)	TAGGGCACTCGCAACAGC
PbFRS10 (OF18405-R)	TGTGGGGATGGGAGTCGT
PbFRS12 (OF07214-F)	GGGATGGAGGGGAGAGCA
PbFRS12 (OF07214-R)	TCCCTCCTCTGCCTTCGT
PbFRS13 (OF28777-F)	GCTGCGCGGGCTTATAGA
PbFRS13 (OF28777-R)	GCAAATGCCGTTGCCCAA
PbFRS16 (OF05523-F)	TGACGTCGGGGTTGAACG
PbFRS16 (OF05523-R)	TCGGCCATTTGCTTTTCCA
PbFRS18 (OF14737-F)	CGGGTGACTCTTCCATGGT
PbFRS18 (OF14737-R)	CAAAACCAGCGGCCCTTG
Internal reference gene (EF1α-F)	CATTCAAGTATGCGTGGGT
Internal reference gene (EF1α-R)	ACGGTGACCAGGAGCA

## Data Availability

Publicly available datasets were analyzed in this study.
